# Low risk of cervical cancer during a long period after negative screening in the Netherlands

**DOI:** 10.1038/sj.bjc.6600843

**Published:** 2003-04-01

**Authors:** M E van den Akker-van Marle, M van Ballegooijen, J D F Habbema

**Affiliations:** 1 Department of Public Health, Erasmus MC, Rotterdam, The Netherlands

**Keywords:** relative risk, cervical cancer, negative screening

## Abstract

A condition for effective cervical cancer screening is a low incidence of cervical cancer after negative screening compared to that in the absence of screening. This relative risk was studied for the period 1994–1997 in the Netherlands and compared with previous studies. All cases of invasive cervical cancer diagnosed from 1994 to 1997 in the Netherlands were related to woman-years at risk, stratified by age, number of preceding negative screenings and time since the preceding negative screening. These incidence rates were compared with that before screening started in the Netherlands. The relative risk increases from 0.13 in the first year after screening to 0.24 after more than 6 years after screening for women with one previous negative screening. These figures reduce to 0.06 and 0.18, respectively, for women with two or more previous screenings. However, these estimates are less favourable when account is taken of the likely decrease in risk for cervical cancer in the period studied. Our data show a low relative risk of cervical cancer for several years following the last negative Pap smear. However, the denominator of the relative risk, that is, the incidence without screening, may have been overestimated. This applies also to the IARC multicountry study, and may have caused too optimistic expectations about the effectiveness of cervical cancer screening.

The effectiveness of cervical cancer screening has never been established by randomised controlled trials. Evidence for mortality reduction, the primary aim of cervical cancer screening, has come from studies that compared regions or individuals with different screening intensities (Clarke and Anderson, 1979; Laara *et al*, 1987; van der Graaf *et al*, 1988). One indicator of such effectiveness is the incidence of cervical cancer after a negative screen related to that in the absence of screening. The smaller this relative risk, the better has screening succeeded in selecting women at low risk of getting cervical cancer in subsequent years. Combined with the improvement in prognosis for women with a true positive screening result, such a selective power warrants a reduction in incidence and mortality.

The present study estimates the relative risk for cervical cancer after a negative screen on the basis of nationwide Dutch data. This risk is determined by the duration of the screen-detectable preclinical stage and the sensitivity of the test for this stage. The predictive value of a negative screen for not developing cervical cancer increases with a longer preclinical duration and a higher sensitivity.

When data from large-scale screening programmes became available, a working group of the International Agency on Research for Cancer (IARC) estimated the incidence after a negative screen, compared to the estimated background incidence in eight countries, that is, Canada, Scotland, Iceland, Denmark, Norway, Sweden, Switzerland and Italy (IARC Working Group, 1986a,1986b). Expectations about the effectiveness of cervical cancer screening are often based on the results of this ‘classic’ study, and important models for such screening have been validated using the IARC results (Eddy, 1987; Gustafsson and Adami, 1990; Gyrd-Hansen *et al*, 1995; van Oortmarssen and Habbema, 1995). This was the rationale for comparing the results of the present study with those of the IARC study: if the results correspond, expectations concerning the effects of cervical cancer screening are reinforced.

## MATERIALS AND METHODS

All cytological and histological examinations of the cervix in the Netherlands up to 31 December 1997 were retrieved from the Pathological National Automated Archive (PALGA). When this registry started in 1975 few laboratories participated, but within a decade a high level of national coverage was achieved. Using the PALGA identification method (i.e. first four characters of the family name, date of birth and gender), different examinations of the same woman could be linked. In this study, all cases of invasive cervical cancer occurring from 1994 to 1997 were identified by selecting histologically confirmed diagnoses of invasive cancer from the database. These include all malignant neoplasms of the cervix, most of which are squamous-cell carcinomas.

Woman-years were counted for each woman, from each negative screen until the next negative screen, until the histological diagnosis of a (precursor of) invasive carcinoma, or until 31 December 1997. A negative screen was defined as an episode consisting of a cytological or histological examination with a negative result, or a cytological examination with a positive result without a histological confirmation of a (precursor of) invasive cancer (see appendix and Table 2

**Table 2:** Definition of primary and secondary examinations

An examination is secondary if in the preceding 48 months:
• there is a histological diagnosis of invasive cervical cancer
• there is a histological diagnosis of preinvasive cancer after which there has not been three cytological examinations with a negative result
• there is a cytological diagnosis of severe dysplasia after which there has not been three cytological examinations with a negative result
• there are at least two cytological diagnoses of light–moderate dysplasia after which there has not been three cytological examinations with a negative result
• there is a histological examination without diagnosis after which there is no histological examination with a negative result or three cytological examinations with a negative result
• there is a cytological diagnosis of light–moderate dysplasia after which there is no cytological examination with a negative result
• there is an inadequate cytological examination after which there has not been a cytological examination with a negative result
• there is a cytological examination without endocervical cells after which there has not been a cytological examination with a negative result
otherwise an examination is primary

in the appendix for definitions). The invasive cases were related to woman-years at risk, and presented as the number of cases per 100 000 woman-years at risk. These incidence rates were stratified by age, number of preceding negative screenings and the interval since the preceding negative screen.

The incidence of invasive cervical cancer was calculated for women aged 35–64 years with one previous negative screen and with two or more previous negative screenings. This method is comparable to that of the IARC study. Next, the relative risk for cervical cancer was calculated by dividing the incidence rate after screening by the incidence in the absence of screening.

Since this background incidence cannot be observed, it had to be estimated indirectly. For this, we used the clinical incidence of invasive cervical cancer in the period 1965–1969, this being the last period before screening was introduced in the Netherlands. National incidence figures were based on incidence data of three regions in the Netherlands (Friesland, The Hague and Rotterdam) covering together 8% of the women in the Netherlands (Central Cancer Registry, 1993). Regional differences in cervical cancer incidence have been accounted for by using the differences between the age-standardised mortality rate of cervical cancer for these three regions and for the entire land for the period 1968–1978 as a proxy.

The identification method used by PALGA (first four characters of the family name, etc.) is not 100% exclusive and will sometimes combine two or more women in one identification code. To investigate the influence of this lack of discriminative power of the identification key, we also calculated the incidence rates excluding the examinations of those women with 0.5 and 1% of the most frequently occurring first four characters of the family name. The corresponding percentages of women thus excluded from analysis are 31.7 and 43.5%, respectively.

The lack of discriminative power of the identification key leads to an upward bias in incidence after a negative screen, because negative screening results may be erroneously linked to a cancer. We indeed found that the incidence rate including all women is about 20% higher after one and two or more negative screenings than the rate after excluding 0.5% of the most frequent first four characters of the family name. As the difference in incidence between excluding 0.5 and 1% of the most frequent first four characters is very small, in our analyses, we chose to exclude only those women with 0.5% of the most frequent first four characters of family name in the corresponding table and figures (Table 1

**Table 1:** Relative risk of cervical cancer [95% confidence interval] after two or more negative screenings over time since the last negative screening for women aged 35–64 years and the number of actual cancer cases (1994–1997)

	**Relative risk**		
**Interval**	**Incidence in 1965–1969**	**Projected incidence^a^ in 1994–1997 in a situation without screening**	**Number of cancer cases**
0–6 months	0.12 [0.08–0.17]	0.26 [0.18–0.39]	26
7–12 months	0.06 [0.03–0.10]	0.14 [0.08–0.23]	13
1–2 years	0.08 [0.06–0.12]	0.19 [0.13–0.26]	31
2–4 years	0.15 [0.11–0.19]	0.33 [0.26–0.42]	65
4–6 years	0.20 [0.14–0.29]	0.45 [0.32–0.64]	31
6–10 years	0.18 [0.11–0.30]	0.41 [0.25–0.68]	15

Two different ways of calculating the background incidence (i.e. the denominator of the relative risk) were used (see text).

a Based on the APC analysis of prescreening mortality (see Discussion).

, Figures 1

**Figure 1:**
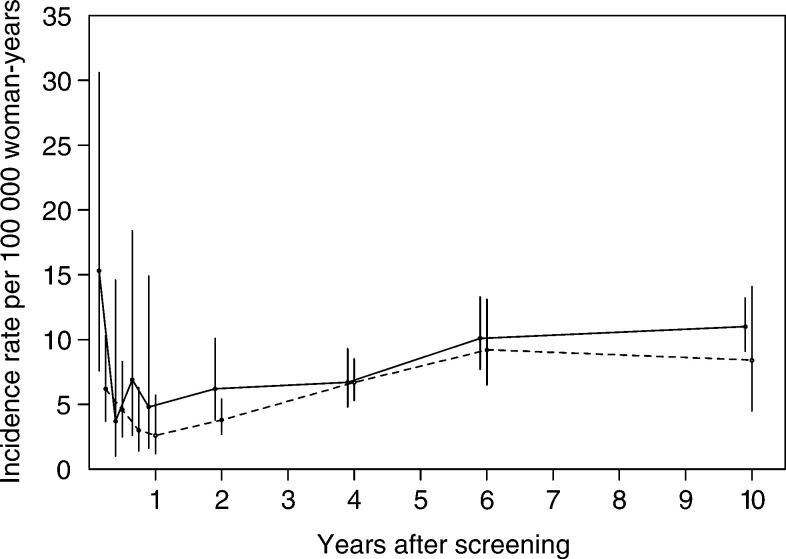
Incidence of invasive cervical cancer over time since the last negative screen, for women with one and two or more preceding negative screenings. The solid line respresents one negative screen and the dashed line represents two or more negative screens (95% CIs are shown).

and 2

**Figure 2:**
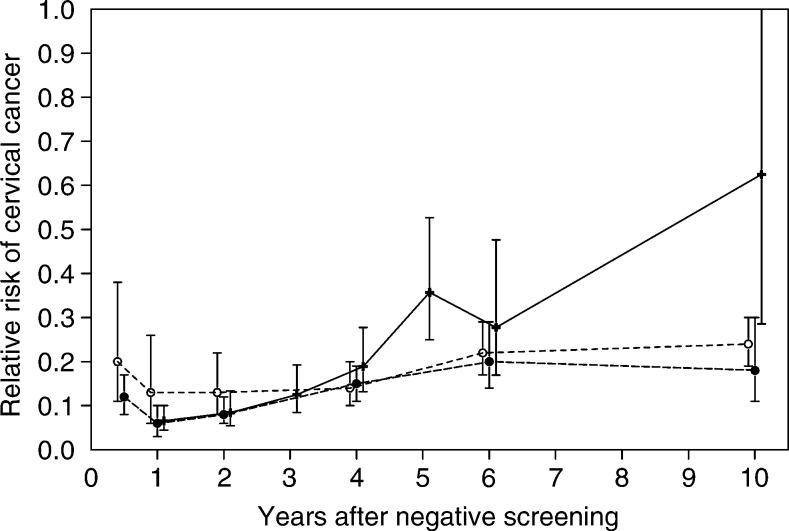
Relative risk of invasive cervical cancer after negative pap smears as assessed in the eight countries contributing to the IARC study (see text), compared with the risk in the Netherlands after one and two or more negative screens (95% CIs are shown). —+—: IARC, ⩾ two negative smears; ---○---: Netherlands, one negative screen; ---•---: Netherlands, ⩾two negative screens.

). This served to limit the lack of discriminative power of the identification key while maintaining sufficiently large numbers on which to base our analysis.

## RESULTS

A total of 1648 invasive carcinomas in women aged 35–64 years were retrieved in the period 1994–1997 in the Netherlands. Of these carcinomas, 879 were diagnosed without a preceding negative screen, 376 after one negative screen and 393 after two or more preceding negative screenings.

Figure 1 shows the incidence of invasive carcinoma per 100 000 woman-years by interval since the last negative screen for women with one and with two or more negative screenings. In the first months after negative screening, the incidence of cervical cancer is relatively high. This may be because the women and/or physicians were not reassured by the recent (false) negative Pap smear result (e.g. because of persisting signs or symptoms) and thus elected for additional diagnostic procedures. After this initial peak, the incidence is low and will mainly consist of cases of neoplasia missed at screening. Over time, the incidence increases because of new lesions that developed after the negative screening.

Figure 2 shows the incidence of invasive cervical cancer over time since the previous negative screening, compared with the incidence of invasive cervical cancer in the period 1965–1969 in the Netherlands, which was 46.1 per 100 000 women-years for women aged 35–64 years. In the first years, the relative risk is lower after two or more negative screenings than after only one negative screening. Figure 2 also compares our incidence data with the IARC results. In the first years after a negative screen, the relative risk is comparable in the two studies, but from 4 years onwards the relative risk is higher in the IARC study than in the Netherlands.

## DISCUSSION

As estimated from an age period cohort (APC) analysis of prescreening mortality rates in the Netherlands, the risk of cervical cancer decreases sharply for cohorts of women born after 1927 (van Ballegooijen, 1998). Based on these data, the projected incidence for the period 1994–1997 in the absence of screening on the basis of these figures was 20.5 per 100 000 woman-years. For the women born after 1950 this might be an underestimate because there was an increased risk for cervical cancer in the youngest cohorts (Beral *et al*, 1994), whereas we extrapolated the low risk of the latest cohort for which prescreening mortality rates were available (born 1940–1950) to the youngest cohorts. Table 1 gives the relative risk using the projected incidence for the period 1994–1997 (from the APC analysis) compared with the incidence just before screening started (1965–1969). Using the projected incidence for 1994–1997 results in a factor two higher risk.

Estimation of the background incidence has also proven problematic in other studies. In the IARC study, some centres used a case–control approach whereas (as in the present study) others used a cohort approach. Some cohort studies used the incidence in women who were never screened as background incidence, while others used the incidence before screening became widespread. Both estimates have their problems: that is, women never attending screening are reported to be at higher risk for cervical cancer (Berget, 1979; Magnus *et al*, 1987; van Oortmarssen and Habbema, 1991) and in several countries a nonscreening-related decrease in cervical cancer risk in the considered period is reported (Laara *et al*, 1987; Cuzick and Boyle, 1988; van Ballegooijen, 1998). The case–control studies contributing to the IARC results may also have resulted in an underestimation of the relative risk because of healthy screenee bias and frequency bias. Sasieni *et al* found a factor two higher relative risk compared with the relative risk of the IARC and the present study. However, they used a case–control approach with carefully selected appropriate controls to cases, which may have reduced the bias (Sasieni *et al*, 1996). Viikki *et al* (1999), who found a three times higher relative risk, used the incidence of the total population in the screening period. However, because of the incidence-reducing effect of screening this will be an underestimation of the background incidence, which may have led to the relatively high risks.

Other differences between studies are less important. The IARC results were presented for two or more previous negative screenings only, whereas other studies (Mitchell and Giles, 1996; Sasieni *et al*, 1996; Viikki *et al*, 1999) also included a single previous screening. This latter case leads to a higher incidence and thus relative risk, especially in the period immediately following a negative screen (see Figure 2).

In contrast to the four studies discussed above, we considered a positive smear result that was not followed by a histological diagnosis as a negative screen. Calculating the relative risk after a negative Pap smear did not have a strong effect on the results.

As a result of the methodological differences, comparison of the performance of screening between different countries is difficult. Nevertheless, for example, the suspected suboptimal performance of screening in the UK in the 1990s (Raffle *et al*, 1995; Anonymous, 1998) may have contributed to the high relative risks reported in that period (Sasieni *et al*, 1996).

In most of the prominent models on cervical cancer screening (Eddy, 1987; Gustafsson and Adami, 1990; Gyrd-Hansen *et al*, 1995; van Oortmarssen and Habbema, 1995), the IARC results have been used to validate the assumptions of the model regarding the sensitivity for, and duration of, the preclinical disease stage. If, however, the IARC results are too favourable (e.g. because of the underestimation of the background risk) then these models will also overestimate the effectiveness of cervical cancer screening.

The longer ago screening was started, the greater the uncertainty will be about the background incidence. As a result, assessment of the relative risk after negative screening will become increasingly difficult, that is, more difficult than in the IARC study and the current study.

In conclusion, our data show that the relative risk for cervical cancer incidence is low for several years following a negative screening using the Pap smear. There are strong indications that relative risk estimates are too favourable, because of a too high estimate of the background incidence. However, even an underestimate of the background incidence shows a considerable reduction in the relative risk after negative screening. The overestimation also applies to the widely used IARC results, and may have raised too optimistic expectations about the effectiveness of cervical cancer screening.

## APPENDIX

### Episodes

After linking all examinations belonging to one woman (based on the first four characters of her family name and date of birth), the examinations were divided into primary and secondary ones. The definition of primary and secondary examinations is given in Table 2. Briefly, an examination is considered to be secondary if in the 48 months preceding the examination there has been a non-negative examination result that could have given rise to the present examination. If not, the examination is a primary one. Next, series of examinations belonging to one woman were divided into episodes. An episode is defined as a time period consisting of a primary examination with (in case the primary examination is not negative) the accompanying follow-up examinations. If a primary examination is negative, the episode consists of that examination only.

### Screen-detected and interval carcinomas

An invasive carcinoma was categorised as screen-detected when, in the episode in which the invasive carcinoma is diagnosed, the reason for the primary smear was coded as being for screening purposes. If the reason for the primary smear was not known, the invasive carcinoma was considered to be screen-detected if no biopsy was taken at the same time as the primary examination and if there was no unexpected (from a follow-up point of view) histological examination in the episode. Otherwise, the invasive carcinoma was considered to be diagnosed because of symptoms.

### Age

Age was determined at the time of the screening examination for screen-detected carcinomas and at the time of histological confirmation of invasive cancer for symptomatic cases.

### Negative examinations

All primary examinations, both cytological and histological, without a histological confirmation of a (precursor of) invasive cancer in their episode, are counted as a negative examination. This implies that if the primary examination had a positive result, this examination is counted as negative examination if no histological confirmation of the positive smear result takes place.

In case an episode contains a histological diagnosis of a cervical neoplasia, we assume that the woman will have the normal risk of getting cervical cancer after the treatment of the cervical abnormality, and the counting of the negative examinations is reset to zero.

### Interval since the preceding negative examination

The interval since the preceding negative examination is defined as the time between the last negative primary examination and the screening examination for screen-detected carcinomas and the time between the last negative primary examination and the histological confirmation of invasive cancer for cancers detected because of symptoms.
